# Effects of crude oil prices on copper and maize prices

**DOI:** 10.1186/s43093-021-00100-w

**Published:** 2021-11-11

**Authors:** Byrne Kaulu

**Affiliations:** 1 Department of Accounting and Finance, School of Business, Copperbelt University, P. O. Box 21692, Kitwe, Zambia; 2 University of Zambia, Lusaka, Zambia

**Keywords:** Cointegration, Granger causality, VAR, VECM, Copper price, Crude oil price, Maize price, Q02, Q43, E64, E66, F41

## Abstract

This study explains the effects of crude oil prices on copper and maize prices. Vector autoregressive and vector error correction models are used to study the relationship between oil prices and prices of copper and maize. The commodity price data used consist of average monthly prices of each of the commodities: crude oil, copper and maize for the months January 1982 to June 2021. For robustness, the analysis was also run on a sample of the same data for the period January 2000 to June 2021. A long-run relationship was found between crude oil and copper prices on the one hand and maize prices on the other for the 1982 to 2021 period at the 5% significance level. The same was not true for the shorter sample (2000 to 2021). Granger causality flowing from crude oil prices alone to copper and maize prices was not found. Recommendations that are useful for energy, mining, agriculture and general development policy and practice are made. The findings are also useful for bilateral and multilateral aid discussions. The limitations of the study and recommendations for future scholarship are also made.

## Introduction

Crude oil, copper and maize are essential to both poor and rich nations. Maize for example is a staple food in vast parts of Sub-Sahara Africa (SSA) and accounts for 40% of all cereals production in that region [11]. It is also a staple food for several countries in Latin America. Copper is used in electronic public defense and military equipment as well as industrial and domestic electronic equipment [6]. Crude oil, sometimes called the ‘black gold,’ is arguably the most significant of the three. It is a basic input in industrial production and is used to fuel cars and planes for transportation and generate electricity for use in production of goods and services. A change in the price of oil is therefore likely to affect several aspects of an economy.

One particular area which can be affected and has been increased global concern in the last decade is volatility of food prices. This concern has found itself on the agendas of the G20 countries [29]. Additionally, the volatility of crude oil prices has been a concern because of the importance of the commodity in mining; for instance, copper mining. This highlights the importance of studies discussing the relationships among the prices of crude oil, copper and maize.

Most studies on crude oil prices, copper prices and maize price are either country specific (see for instance [29]) or are too general by covering effects of crude oil prices on other commodity prices. They often decompose variation in relation to several other commodity prices in the economy [10, 36]. In order to highlight the relationships among crude oil price, copper price and maize price specifically and influence policy in these particular areas, this study concentrates on the three commodities (crude oil, copper and maize) with the assumption of ceteris paribus. Maize is chosen because it is a staple food for many countries in SSA and Latin America. It is also used as a supplement in food products in other parts of the world. Copper is chosen because of its significance in everyday life globally. It is used as an alloy in jewelry, for medical purposes [28], and copper wires are used in domestic and industrial electronic devices world over. Using data from the World Bank, this study attempts to answer the following questions:

1. Do maize prices, copper prices and crude oil prices have long-run relationships?
2. What are the causal relationships among maize prices, copper prices and crude oil prices?
3. Can crude oil price specifically cause volatility in the prices of other two commodities?

Answering these questions is important for multilateral and bilateral policy. For instance, it can inform better formulation of Bilateral Investment Treaties (BITs). Some countries in the world are net exporters of crude oil, while others are net importers. The same is true for copper and maize. While net importers of crude oil usually suffer negative consequences from oil price rises [1, 8], some have other natural resources (like copper) they can leverage on for stability. Agriculture is also one industry that nations can leverage on for improved economic stability. Understanding the causal relationships among the prices of commodities in the different sectors can help respective nations discuss bilateral and multilateral agreements from an informed perspective.

## Literature review

### Theoretical review

Modern agriculture and mining use products from oil to fuel machinery in farms and mining plants, respectively. They also use them to fuel transportation of output from the production centers to the markets. For this reason, high and volatile crude oil prices are of concern in the mining and agriculture sectors of the economy.

Several studies have attempted to explain the effects of crude oil prices on metal prices [18, 34] and food prices [29, 38]. However, at the moment, no single theory explains the effects of crude oil prices on commodities in general [35].

The Dutch disease is one theory advanced in the literature to explain why resource-rich nations (for example, oil-rich nations) remain poor. In this theory, the more money a nation makes from its mining activities, the less competitive other industries become. Investments in mining become lucrative, and investors move funds from other industries like farming to mining. Eventually, the rest of the industries fail to flourish. The main stream theory of economic growth posits that economic growth is a function of production and production requires energy [20]. In this theory, land, labor and capital are primary factors of production and energy resources are intermediate inputs. The ideal price of crude oil according to this theory is the marginal product of its output. Another theory, the linear or symmetric theory of economic growth [16, 17] argues that the price of oil drives volatility in the growth rate of output and this relationship is inverse in nature. A theory of the asymmetric effect of oil prices however suggests that oil prices have varying effects on production [2].

While the theories are useful for explaining the relationship between oil prices and economic growth, they concentrate on output as an outcome rather than price. They can directly explain the influence of oil prices on maize and copper production but not prices. To bring in an aspect of price, basic demand and supply theory can be used in conjunction with the above theories. For instance, as per symmetric theory of economic growth, higher oil prices would lead to lower production of copper and maize. In line with demand and supply theory, lower copper and maize output would lead to higher prices of these commodities. Therefore, theoretically, higher oil prices are likely to lead to higher copper prices and maize prices. This is the point of departure for which empirical evidence is used to test theory in this article.

### Empirical review

The relationship between energy and commodity prices in general has been the focus of many studies. Some literature supports the notion that the markets are mostly not dependent [27, 38], while others conclude that there is at least some strong unidirectional relationship [4, 13]. However, the effect crude oil prices have on copper and maize prices together is largely understudied. This study therefore seeks to fill this contextual gap while contributing to literature on the effects of energy prices on commodity prices (the context of crude oil, copper and maize).

Jiang et al. [21] studied volatility spill overs in the US crude oil, maize and plastic markets. The data set used consisted of 393 weekly observations from July 31, 2006, to February 6, 2014. They studied the price transmission mechanism by estimating a VECM. They found that the price of oil transmits volatility to maize prices but not to the plastics market. While this study had two variables of interest, it left out copper—our other variable of concern in this study.

Nwoko et al. [29] studied the effects of crude oil prices on the volatility of maize prices in Nigeria using annual data for the year 2000 to the year 2013. Cointegration, VECM modeling and Granger causality tests were used in the analysis. Unidirectional causality running from oil price to the food price volatility was found. A long-run relationship was found. Additionally, a positive short-run relationship between crude oil price and food price volatility was found.

Roman et al. [33] studied the connection between crude oil prices and five food price indices. Data from 1990 to 2020 and the VECM were used to achieve the objectives. A long-run relationship between crude oil prices and the meat index was found. A relationship between crude oil prices and the cereals price index was only found for the short run.

Nazlioglu and Soytas [27] investigated the effects of oil prices on agricultural commodity prices in Turkey. This study employed the Toda–Yamamoto causality approach and generalized impulse response analysis to study monthly data in the range January 1994 to March 2010. They found that oil prices have neither a direct nor indirect effect on individual agricultural prices in Turkey, through the exchange rates. Maize was one of the agriculture products included in the study.

Peša [30] studied the relationship between crops and copper for the period 1950 to 2000 on the Copperbelt region of Zambia and Democratic Republic of Congo. This qualitative study found that falling copper prices in the 1970s led to the government of Zambia calling for ‘back to the land’ policies. This involved an ‘agrarian revolution’ approach where more people were encouraged to farm in order to ensure food security.

Baek and Koo [4] studied the predictors of food price inflation in the USA. They conducted cointegration analysis on data from 1991 to 2008. They found that energy prices affect food prices. They also highlight that because of recent trends toward the use of crop-based biofuels, the price of maize is closely linked with the price of crude oil. While this study provides great general insights, it does not specifically address the effect of crude oil prices on maize price in the context of maize being a staple food for many nations.

Given the foregoing studies, it is hypothesized that:

#### H_1_

Crude oil prices cause maize prices

#### H_2_

Crude oil prices have a long-run relationship with maize prices

#### H_3_

Crude oil prices have a short-run relationship with maize prices

Maitra et al. [26] analyzed the asymmetric volatility connectedness between oil and commodities. They found that copper is a net transmitter of volatility to oil and other markets. They also found that among the variables they studied, crude oil was a net receiver of volatility. They also found that copper receives volatility from crude oil markets. The limitation of this study is that it focused on optimal portfolio selection for investors rather than general economic policy recommendations.

Ezeaku et al. [12] studied the volatility of commodity prices during the COVID-19 pandemic using daily commodity price data from December 2, 2019, to October 1, 2020. Structural vector autoregressive (SVAR) modeling was used for data analysis. Impulse responses in this study showed that copper prices responded positively to crude oil price shocks for 130 days and then negatively for the rest of the time. This is similar to Chen and Saghaian [7] who found strong relationships between crude oil prices and commodities during the 2008 global financial crisis.

A study by Kaushik [22] investigates the effects of global crude oil prices on metal prices in India. Dynamic conditional correlation generalized autoregressive conditional heteroskedasticity modeling was applied on data from June 1, 2006, to March 31, 2017. While several other metals were studied, the price of copper was found to have a weak positive correlation with the crude oil price. The authors posit that the same global economic factors that drive crude oil prices also drive copper prices.

Zhang and Tu [37] studied the effects that global oil price shocks have on China's metal markets with a focus on copper and aluminum. Autoregressive conditional jump intensity (ARJI) and model, combining with the generalized conditional heteroskedasticity models, was used for data analysis. Crude oil price shocks were found to have significant symmetric impacts on the metal markets.

It is therefore hypothesized that:

#### H_4_

Crude oil prices cause copper prices

#### H_5_

Crude oil prices have a long-run relationship with copper prices

#### H_6_

Crude oil prices have a short-run relationship with copper prices

## Methods

### The data

Secondary time series data were used in this study. Monthly commodity price data for about four decades from January 1982 to June 2021 were obtained from the WB. Crude oil price data in this study consist of monthly averages of three crude oil prices—representative of world prices. These are the Brent crude oil price, Dubai crude oil price and the West Texas Intermediate (WTI) crude oil price. Maize prices are those of yellow maize; free on board (FOB) as at the gulf ports in the USA. These are the closest proxies for world commodity prices for the respective commodities that could be found in the WB dataset. The London Metal Exchange (LME) settlement prices are the copper prices used.

### Data analysis

For robustness, data analysis was done on data from January 1982 to June 2021 (the whole available data set) and then also run on data for the latest twenty years (2000 to 2021). Descriptive as well as inferential analysis was conducted. Descriptive statistics included kurtosis, skewness as well as Jarque–Bera test of normality, mean and standard deviation. The movements of the variables are illustrated using graphs. Optimal lag selection criteria were used to select an optimal lag. The Augmented Dickey Fuller (ADF) test was used to check for stationarity. The long-run relationships among the variables were checked using Johansen’s cointegration test, while short-run tests were done using the vector autoregression (VAR) and the vector error correction model (VECM). The latter were used based on the results from the cointegration test. Impulse response functions (IRF) and variance decomposition functions (VDF) were used to describe the movement of each variable in relation to the other two. Causal relationships were tested using Granger causality tests.

#### Unit root tests

Unit root tests are used to check for stationarity. A variable is stationary if its mean, variance or both are constant with time [19]. The absence of stationarity leads to spurious regressions (see [15]). That is why it is important to check for stationarity before proceeding to do the relevant time series analysis. The ADF test, popularized by Dickey and Fuller [9], is the most commonly used test for stationarity [29]. It is therefore adopted for this study.

#### Cointegration

A cointegration test is utilized to establish whether there is a long-run relationship or correlation between time series. Several ways of testing for long-run relationships (cointegration) between variables exist. Three major tests used are the Engle–Granger two-step method, Johansen test and the maximum eigenvalue test. Most extant literature [4, 21] uses the Johansen cointegration test. It is therefore the main method adopted in this study. If the variables are not co-integrated, the vector autoregression (VAR) model is run. When they are co-integrated, a vector error correction model (VECM) is recommended [3].

#### Vector error correction model (VECM)

The use of VECM is based on the outcome of cointegration tests and is in line with extant literature [5, 29]. In addition to explaining short-run relationships, this model helps illustrate how deviations from equilibrium in the long-run model are adjusted for in the short run. Equations 1, 2 and 3 illustrate the model used in this study on the data from 1982 to 2021.

1 $$\Delta {\text{Copper}}_{t} =\, a + \sum\limits_{i = 1}^{k - 1} {B_{i} \Delta {\text{Copper}}_{t - i} } + \sum\limits_{j = 1}^{k - 1} {\phi_{j} \Delta {\text{Maize}}_{t - j} } + \sum\limits_{j = 1}^{k - 1} {\varphi_{m} \Delta {\text{Oil}}_{t - m} + \lambda_{1} {\text{ECT}}_{t - 1} + u_{1t} }$$

2 $$\Delta {\text{Maize}}_{t} =\, \sigma + \sum\limits_{i = 1}^{k - 1} {B_{i} \Delta {\text{Copper}}_{t - i} } + \sum\limits_{j = 1}^{k - 1} {\phi_{j} \Delta {\text{Maize}}_{t - j} } + \sum\limits_{j = 1}^{k - 1} {\varphi_{m} \Delta {\text{Oil}}_{t - m} + \lambda_{2} {\text{ECT}}_{t - 1} + u_{2t} }$$

3 $$\Delta {\text{Oil}}_{t} =\, \vartheta + \sum\limits_{i = 1}^{k - 1} {B_{i} \Delta {\text{Copper}}_{t - i} } + \sum\limits_{j = 1}^{k - 1} {\phi_{j} \Delta {\text{Maize}}_{t - j} } + \sum\limits_{j = 1}^{k - 1} {\varphi_{m} \Delta {\text{Oil}}_{t - m} + \lambda_{3} {\text{ECT}}_{t - 1} + u_{3t} }$$

where ${\text{Copper}}_{t - i}$ = lagged copper price, ${\text{Maize}}_{t - j}$ = lagged maize price, ${\text{Oil}}_{t - m}$ = lagged crude oil price, $t$ = time (months), $u_{1t}$, $u_{2t}$ and $u_{3t}$ = shocks/impulses, innovations, ${\text{ECT}}$ = error correction term and *λ* = speed of adjustment.

#### Vector autoregressive (VAR) model

The sample from 2000 to 2021 required use of the VAR model. Extant literature [39] uses this model when there is no evidence of cointegration. Equations 4, 5 and 6 provide the specification.

4 $${\text{Copper}}_{t} =\, a + \sum\limits_{i = 1}^{k} {B_{i} {\text{Copper}}_{t - i} } + \sum\limits_{j = 1}^{k} {\phi_{j} {\text{Mainze}}_{t - j} } + \sum\limits_{j = 1}^{k} {\varphi_{m} {\text{Oil}}_{t - m} + u_{1t} }$$

5 $${\text{Mainze}}_{t} =\, \sigma + \sum\limits_{i = 1}^{k} {B_{i} {\text{Copper}}_{t - i} } + \sum\limits_{j = 1}^{k} {\phi_{j} {\text{Mainze}}_{t - j} } + \sum\limits_{j = 1}^{k} {\varphi_{m} {\text{Oil}}_{t - m} + u_{2t} }$$

6 $$Oil_{t} =\, \vartheta + \sum\limits_{i = 1}^{k} {B_{i} {\text{Copper}}_{t - i} } + \sum\limits_{j = 1}^{k} {\phi_{j} {\text{Mainze}}_{t - j} } + \sum\limits_{j = 1}^{k} {\varphi_{m} {\text{Oil}}_{t - m} + u_{3t} }$$

where ${\text{Copper}}_{t - i}$ = lagged copper price ${\text{Mainze}}_{t - j}$ = lagged maize price, ${\text{Oil}}_{t - m}$ = lagged crude oil price $t$ = time (months), $u_{1t}$, $u_{2t}$ and $u_{3t}$ = shocks.

#### Causality

In order to understand any bidirectional or unidirectional causal relationships, Granger causality [14] was used.

## Results

### Descriptive statistics

**Fig. 1:**
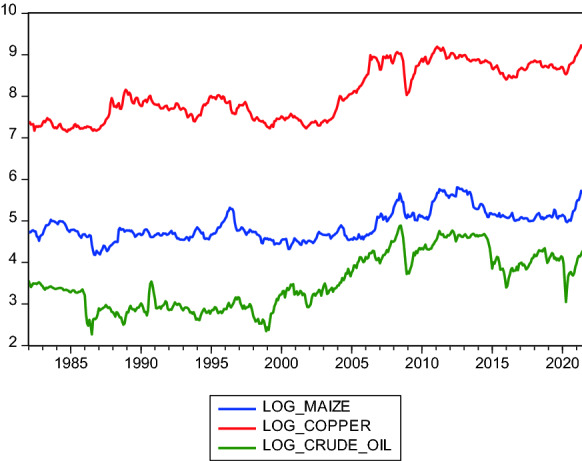
Maize, copper and crude oil price trends 1982—2021

The trends of the logs of the maize prices, copper prices and crude oil prices for the months from January 1982 to June 2021 are shown in Fig. 1. A visual inspection suggests that the crude oil prices and maize prices move closely together over time. All three variables are relatively stationary. The most fluctuations and troughs are observable in the 1980s (likely due to the great depression), mid-2000s (likely due to the global financial crisis) and early 2020 when COVID-19 was pronounced a pandemic by the World Health Organization.

**Fig. 2:**
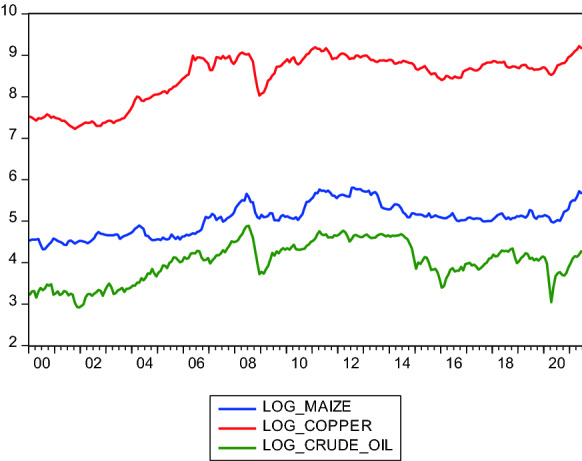
Trend of crude oil, copper and maize prices (2000 to 2021)

Figure 2 shows the trend for January 2000 to June 2021. A visual inspection suggests that the log of crude oil prices and that of maize prices move closely together. It also confirms the troughs around 2007–2008 (during the global financial crisis) and in 2020 when COVID-19 became a pandemic.

Descriptive statistics for 1982 to 2021 are shown in Table 1. Additionally, the skewness, kurtosis and Jarque–Bera tests of normality are shown. The Jarque–Bera test suggests that the normality assumption was not met. However, the skewness and kurtosis figures all fall within the range of ± 3. This indicates that residuals are approximately normally distributed. Normality is a desirable characteristic for the data analysis required. All the variables were positively skewed implying that the markets for maize, copper and crude oil have potential for small frequent losses and large infrequent gains. All variables were platykurtic [23, 24].

**Table 1:** Maize, copper and crude oil price trends 1982–2021

	LOG_MAIZE	LOG_COPPER	LOG_CRUDE_OIL
Mean	4.8892	8.0664	3.5375
Median	4.7841	7.9159	3.3906
Maximum	5.8083	9.2264	4.8890
Minimum	4.1791	7.1491	2.2635
SD	0.3557	0.6405	0.6605
Skewness	0.6657	0.2220	0.2788
Kurtosis	2.8672	1.5294	1.8509
Jarque–Bera	35.3521	46.6083	32.2197
Probability	0.0000	0.0000	0.0000

The descriptive statistics for January 2000 to June 2021 are shown in Table 2. The results generally tell the same picture as those for the years 1982 to 2021.

**Table 2:** Descriptive statistics 2000 to 2021 data

	LOG_MAIZE	LOG_COPPER	LOG_CRUDE_OIL
Mean	5.0537	8.4711	4.0139
Median	5.0900	8.7036	4.0765
Maximum	5.8083	9.2264	4.8890
Minimum	4.3211	7.2279	2.9188
SD	0.3744	0.5757	0.4855
Skewness	0.1136	-0.9314	-0.2867
Kurtosis	2.2258	2.4548	2.1095
Jarque–Bera	6.9978	40.5004	12.0601
Probability	0.0302	0.0000	0.0024

### Unit root test

Table 3 shows the results of the ADF test for stationarity. All the three variables (maize prices, copper prices and crude oil prices) were not stationary at level but became stationary at first difference (integrated of order 1). This confirmed that a VAR or VECM model can be run.

**Table 3:** Results of ADF test for unit root 1982–2021 data

	With constant	With constant and trend	Without constant and trend
	*t*-statistic (*p* value)	*t*-statistic (*p* value)	*t*-statistic (*p* value)
LOG_MAIZE	− 1.9124 (0.3265)	− 2.9578 (0.1455)	0.4227 (0.8044)
LOG_COPPER	− 1.1693 (0.689)	− 2.4639 (0.3461)	0.9403 (0.9079)
LOG_CRUDE_OIL	− 1.6902 (0.4356)	− 2.9765 (0.1400)	0.0494 (0.6981)
d(LOG_MAIZE)	− 16.4611*** (0.000)	− 16.4612*** (0.000)	− 16.4635*** (0.000)
d(LOG_COPPER)	− 6.0474*** (0.000)	− 6.0452*** (0.000)	− 5.9588*** (0.000)
d(LOG_CRUDE_OIL)	− 14.2595*** (0.000)	− 14.2537*** (0.000)	− 14.2684*** (0.000)
**Result**	**I(1)**	**I(1)**	**1(1)**

(***) Significant at the 1% and *t* value without ***not significant, b: lag length based on AIC, probability based on MacKinnon [25] one-sided *p *values. I(1) Integrated of order 1

The results of the ADF tests for the 2000 to 2021 sample are shown in Table 4. As per 1982 to 2021 data, all the three variables are not stationary at level but become stationary after first difference.

**Table 4:** Results of ADF test for stationarity 2000 to 2021 data

	With constant	With constant and trend	Without constant and trend
	*t*-statistic (*p* value)	*t*-statistic (*p *value)	*t*-statistic (*p* value)
LOG_MAIZE	− 1.572 (0.4955)	− 2.0804 (0.5538)	0.8585 (0.8947)
LOG_COPPER	− 1.6859 (0.4372)	− 2.0531 (0.5690)	0.8882 (0.8995)
LOG_CRUDE_OIL	− 2.2859 (0.1773)	− 2.3329 (0.4142)	0.2805 (0.7665)
d(LOG_MAIZE)	− 3.2940** (0.0162)	− 3.2537* (0.0765)	− 3.1854*** (0.0015)
d(LOG_COPPER)	− 10.1516*** (0.0000)	− 10.1394*** (0.0000)	− 10.1017*** (0.0000)
d(LOG_CRUDE_OIL)	− 10.623*** (0.0000)	− 10.6157*** (0.0000)	− 10.6228*** (0.0000)
**Result**	**I(1)**	**I(1)**	**I(1)**

(***, **, *) Significant at 1%, 5% and 10% level, respectively. If t value is without *not significant, b: lag length based on AIC, probability based on MacKinnon [25] one-sided *p* values. I(1) integrated of order 1

### Optimal lag selection

In order to balance between parsimony and explanatory power [31] in the model, an optimal lag length has to be chosen. The Schwarz's Bayesian information criterion (SBIC) and Hannan-Quinn information criterion (HQIC) showed that the optimal lag is two. The final prediction error (FPE) and Akaike's information criterion (AIC) showed that the optimal lag is three. Table 5 shows the results.

**Table 5:** Optimal lag selection 1982 to 2021 data

Lag	LogL	LR	FPE	AIC	SC	HQ
0	− 554.07	NA	0.00219	2.39084	2.41752	2.40134
1	1799.2	4666.21	9.36E−08	− 7.67056	− 7.56384	− 7.62856
2	1875.7	150.659	7.01E−08	− 7.96017	− 7.773410*	− 7.886664*
3	1886.4	20.8806	6.96e−08*	− 7.967329*	− 7.70054	− 7.86233
4	1889.3	5.64184	7.14E−08	− 7.94116	− 7.59433	− 7.80466
5	1892.8	6.87335	7.31E−08	− 7.9178	− 7.49094	− 7.7498
6	1905.8	24.7683	7.19E−08	− 7.93459	− 7.42768	− 7.73509
7	1916.6	20.73668*	7.13E−08	− 7.94267	− 7.35572	− 7.71166
8	1921.7	9.58511	7.25E−08	− 7.92577	− 7.25879	− 7.66327

*Implies selection of the lag order by the criterion

LR sequential modified LR test statistic (each test at 5% level)

FPE = Final prediction error

AIC = Akaike information criterion

SC = Schwarz information criterion

HQ = Hannan–Quinn information criterion

Based on its wide use in the literature [32], the AIC was used as a basis for optimal lag selection. It is envisaged that this is likely to increase the comparability of the findings.

Table 6 shows the results of optimal lag selection for the 2000 to 2021 data. All the major information criteria confirmed an optimal lag of 2.

**Table 6:** Lag length selection 2000 to 2021 data

Lag	LogL	LR	FPE	AIC	SC	HQ
0	− 163.2155	NA	0.0007	1.2885	1.3298	1.3051
1	989.1006	2268.9020	0.0000	− 7.5744	− 7.4092	− 7.5080
2	1030.5740	80.6964	8.01e− 08*	− 7.8262*	− 7.5370*	− 7.7099*
3	1038.2230	14.7056	0.0000	− 7.8157	− 7.4026	− 7.6496
4	1042.2270	7.6033	0.0000	− 7.7770	− 7.2399	− 7.5610
5	1044.5520	4.3619	0.0000	− 7.7252	− 7.0642	− 7.4594
6	1051.7850	13.4006	0.0000	− 7.7115	− 6.9266	− 7.3959
7	1061.3090	17.4246*	0.0000	− 7.7156	− 6.8067	− 7.3501
8	1064.9990	6.6647	0.0000	− 7.6744	− 6.6416	− 7.2591

*Implies selection of the lag order by the criterion

LR sequential modified LR test statistic (each test at 5% level)

FPE = Final prediction error

AIC = Akaike information criterion

SC = Schwarz information criterion

HQ = Hannan–Quinn information criterion

### Cointegration test

The results of the cointegration test for the 1982 to 2021 data are shown in Table 7. The test was carried out with intercept but no trend specification. The maximum eigenvalue test and the trace statistic test, both, indicate that there is one cointegration relationship in the model. This confirms the presence of a long-run relationship.

**Table 7:** Cointegration test results 1982 to 2021 data

Trace test				Maximum eigenvalue test			
H_0_	H_1_	Statistic	5% critical value	H_0_	H_1_	Statistic	5% critical value
*r* = 0*	*r* ≥ 1	41.4757	35.1928	*r* = 0*	*r* ≥ 1	26.4277	22.2996
*r* ≤ 1	*r* ≥ 2	15.0481	20.2618	*r* ≤ 1	*r* ≥ 2	12.5039	15.8921
*r* ≤ 2	*r* ≥ 3	2.5441	9.1645	*r* ≤ 2	*r* ≥ 3	2.5441	9.1645

Trace test specifies one cointegration relationship (at *p* = 0.05)

Max-eigenvalue test confirms one cointegration relationship (at *p* = 0.05)

*means rejection of the hypothesis (at *p* = 0.05)

The same analysis was carried out on the January 2000 to June 2021 sample. The test was carried out with intercept but no trend specification in order to maintain consistency with the specification for the 1982 to 2021 data. Both the trace statistic test and the eigenvalue test show that there is no cointegration, at 5% significance. Table 8 shows the results).

**Table 8:** Cointegration test results 2000 to 2021 data

Trace test				Maximum eigenvalue test			
H_0_	H_1_	Statistic	5% critical value	H_0_	H_1_	Statistic	5% critical value
*r* = 0	*r* ≥ 1	27.4408	29.7971	*r* = 0	*r* ≥ 1	12.5546	21.1316
*r* ≤ 1	*r* ≥ 2	14.8861	15.4947	*r* ≤ 1	*r* ≥ 2	11.6288	14.2646
*r* ≤ 2	*r* ≥ 3	3.2573	3.8415	*r* ≤ 2	*r* ≥ 3	3.2573	3.8415

Trace test specifies no cointegration relationship (at *p* = 0.05)

Max-eigenvalue test confirms no cointegration relationship (at *p* = 0.05)

*Means rejection of the hypothesis (at *p* = 0.05)

### Analysis of the long run

One normalized cointegration equation was found in the model for the 1982 to 2021 data. This is shown by Eq. 7 (Table 9).

**Table 9:** Long-run effects of crude oil and copper prices on maize prices

LOG_MAIZE(-1)	1.0000
LOG_COPPER(-1)	− 0.2280
	− 0.1125
	[− 2.0268]
LOG_CRUDE_OIL(-1)	− 0.2749
	− 0.1080
	[− 2.5440]
C	− 2.1047
	− 0.6181
	[− 3.4053]

Standard errors in () & *t*-statistics in []

Equation 1: Long Run Equation

According to the equation, in the long run, increases in copper prices increase maize prices (*β* = 0.2280, SE = 0.1125) and the result is statistically significant (|*t*|= β/SE > 1.96); all else being equal. Further, increases in crude oil prices increase maize prices (*β* = 0.2749, SE = 0.1080) and this result is also statistically significant (|*t*| > 1.96), ceteris paribus. Finally, the constant (2.0147) is also statistically significant (SE = 0.6181).

### Analysis of the short run

#### Speed of adjustment (1982 to 2021 data)

The adjustment coefficient in the relationship between maize prices and the other two commodities (copper prices and crude oil prices) is − 0.0572. This implies that 5.72% of deviations from long-run equilibrium are adjusted for in the short run. This result is statistically significant ((|*t*|= 4.3 > 1.96). The model had an *R*^2^ of 0.1264 and an adjusted R^2^ of 0.1093. This means that 10.93% to 12.64% of variations in maize prices were accounted for by variations in copper prices and crude oil prices in the period January 1982 to June 2021. The results of the other two commodities are also presented in Table 10 for information only.

**Table 10:** Short run VECM estimates

Error correction	D (LOG_MAIZE)	D (LOG_COPPER)	D (LOG_CRUDE_OIL)
Adjustment coefficient	− 0.057213	− 0.026619	0.032668
	− 0.01331	− 0.0137	− 0.02062
	[-4.30001]	[− 1.94240]	[ 1.58401]
D(LOG_MAIZE(-1))	0.288687	0.013741	− 0.057286
	− 0.04642	− 0.04781	− 0.07195
	[ 6.21957]	[ 0.28741]	[− 0.79623]
D(LOG_MAIZE(-2))	− 0.015825	0.01032	0.055682
	− 0.04796	− 0.0494	− 0.07434
	[− 0.32997]	[ 0.20892]	[ 0.74904]
D(LOG_MAIZE(-3))	0.062202	0.040101	− 0.058656
	− 0.04654	− 0.04793	− 0.07213
	[ 1.33660]	[ 0.83661]	[− 0.81314]
D(LOG_COPPER(-1))	− 0.021635	0.367041	0.165486
	− 0.04775	− 0.04919	− 0.07402
	[− 0.45305]	[ 7.46225]	[ 2.23567]
D(LOG_COPPER(-2))	0.099087	− 0.061066	0.201995
	− 0.05038	− 0.05189	− 0.07808
	[ 1.96694]	[− 1.17691]	[ 2.58687]
D(LOG_COPPER(-3))	− 0.016105	− 0.055621	− 0.040205
	− 0.04863	− 0.05009	− 0.07538
	[− 0.33117]	[− 1.11046]	[− 0.53338]
D(LOG_CRUDE_OIL(-1))	− 0.033061	0.055472	0.296999
	− 0.03116	− 0.0321	− 0.0483
	[− 1.06091]	[1.72821]	[6.14853]
D(LOG_CRUDE_OIL(-2))	0.003209	− 0.041581	− 0.138526
	− 0.03205	− 0.03301	− 0.04968
	[ 0.10010]	[− 1.25949]	[-2.78817]
D(LOG_CRUDE_OIL(-3))	− 0.00993	− 0.004262	− 0.024755
	− 0.03106	− 0.03199	− 0.04814
	[− 0.31976]	[− 0.13325]	[− 0.51427]
R-squared	0.12637	0.158528	0.1409
Adj. R-squared	0.109277	0.142064	0.124091

Standard errors in () & t-statistics in []

#### Impulse responses

In order to show the persistent effects of shocks between two prices, impulse response functions (IRF) with Cholesky decomposition are used.

**Fig. 3:**
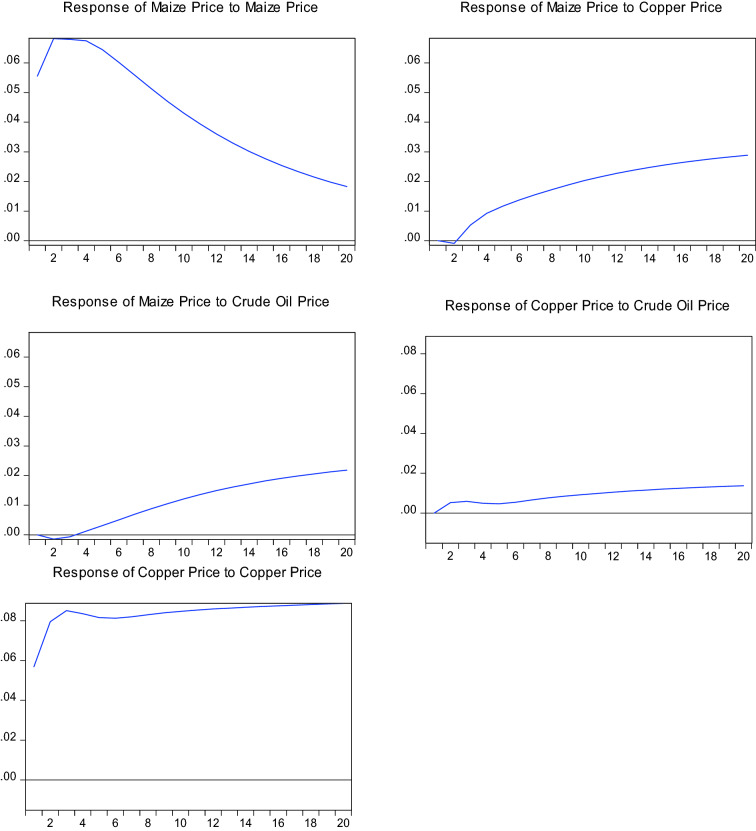
Response of maize prices and copper prices to shocks from crude oil prices 1982 to 2021

The relevant IRFs for the period 1982 to 2021 are shown in Fig. 3. A standard deviation shock in the price of maize is likely to lead to a persistent fall in the price of maize. A standard deviation shock in the price of copper is likely to lead to a persistent rise in the price of maize. A standard deviation shock in the price of crude oil leads to a fall in the price of maize within the first two months. However, this is followed by a persistent rise in price. A standard deviation shock in the price of crude oil leads to a small rise in the price of copper. The latter then almost stabilizes at a slight higher price level. A standard deviation shock in the price of copper leads to a sharp rise in copper prices. Similar to crude oil price shocks, a new higher price level is achieved eventually.

**Fig. 4:**
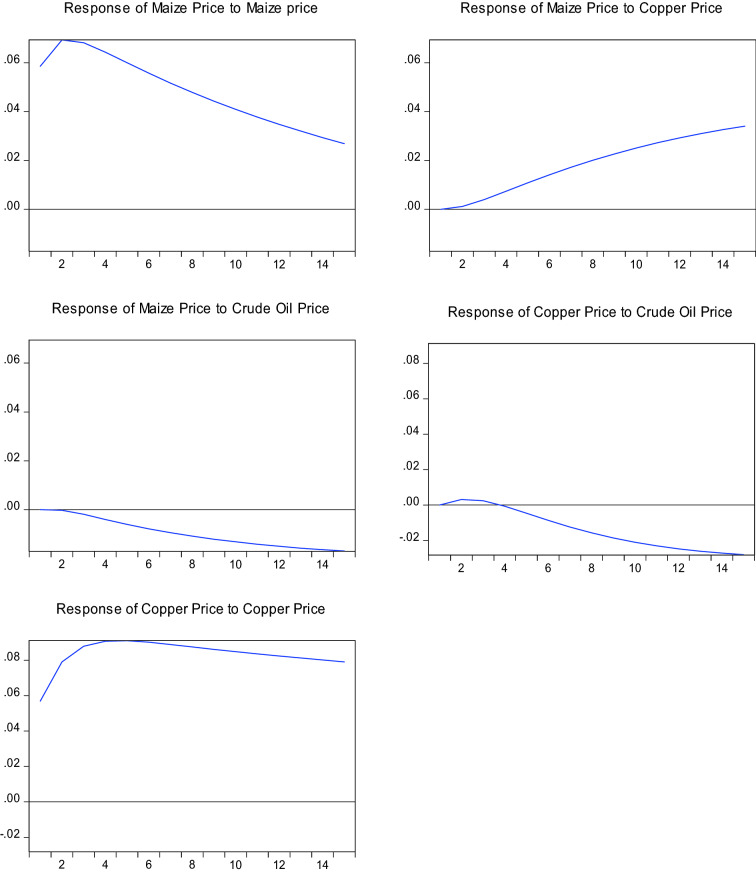
Response of maize prices and copper prices to shocks from crude oil prices 2000 to 2021

When the analysis is run on the data from 2000 to 2021, the results shown in Fig. 4 are obtained. The results are similar to those for 1982 to 2021 except for the response of copper prices and maize prices to shocks from crude oil prices. Unlike the 1982 to 2021 period, the 2000 to 2021 sample suggests that a standard deviation shock in the price of crude oil leads to a persistent fall in the price of maize. A standard deviation shock in the crude oil price causes a slight rise in the copper price in the first two months. Thereafter, the price of copper starts plummeting.

#### Variance decompositions

The variance decomposition (shown in Table 11 for 1982 to 2021 data) shows how much of the variability in a variable is explained by its own shocks in relation to the shocks in other variables in the system. Most public and private sector organizations have a twelve-month budgeting or financial period. Therefore, decompositions are done for six-month intervals with a two-year horizon in order to help policy makers and practitioners make informed decisions.

**Table 11:** Variance decomposition of maize and copper prices—1982 to 2021 data

Equation	Period	SE	Maize price	Copper price	Crude oil price
Maize Price	1	0.06	100.00	0.00	0.00
	6	0.16	98.10	1.74	0.15
	12	0.20	91.30	6.67	2.03
	18	0.23	81.19	13.17	5.64
	24	0.25	70.76	19.53	9.71
Copper Price	1	0.06	1.33	98.67	0.00
	6	0.19	0.86	98.78	0.36
	12	0.28	0.52	98.74	0.74
	18	0.36	0.91	97.93	1.16
	24	0.42	1.50	97.00	1.49

Table 11 shows that maize prices contributed 100% of the variation in their own prices in the first month. In the 6th to the 24th month, the maize price contributed falling percentages ranging from 98.10% and 70.76%, respectively, to its own variation. The price of copper contributed increasing amounts to the variation in the price of maize—going as high as 19.53% in the 24th month. Crude oil prices contribute 0% in month one, and this rises steadily to 9.71% by the 24th month.

The copper price contributed 98.67% to its own variation in the first month. Maize prices contributed 1.33% to the variations in copper prices. By month 24, the copper price contributed 97.00% to its own variation. The contribution of maize to the copper price rose slightly to 1.50%. Interestingly, between months 1 and 24, the contribution of the maize price to the variation in the price of copper falls below 1% before rising back to 1.5%, while that of crude oil rose steadily from 0% in month one to 1.49% in month 24. Copper prices still had a strong influence on their own prices with a 97% contribution to the variation.

Table 12 shows the variance decompositions for the 2000 to 2021 sample. This period was characterized by perilous times in the global commodity markets. Apart from the global financial crises in the early 2000s, the COVID-19 pandemic fell upon the world population. In this period (2000 to 2021) copper prices had a higher influence (30.98% by month 24) on maize prices than they had in the 1982 to 2021 period (7.5% by month 24). The variance decompositions also show that up to 7.32% of variations in copper prices are explained by variations in crude oil prices by month 24.

**Table 12:** Variance decomposition of maize, copper and crude oil prices (2000 to 2021 data)

Equation	Period	SE	Maize price	Copper price	Crude oil price
Maize price	1	0.06	100.00	0.00	0.00
	6	0.16	97.90	1.62	0.49
	12	0.20	87.69	9.64	2.67
	18	0.23	73.92	20.76	5.32
	24	0.26	61.52	30.98	7.50
Copper price	1	0.06	3.48	96.52	0.00
	6	0.21	4.07	95.68	0.25
	12	0.30	3.14	94.19	2.68
	18	0.36	2.30	92.32	5.39
	24	0.41	1.81	90.88	7.32
Crude oil price	1	0.09	2.22	13.72	84.06
	6	0.25	8.24	35.21	56.55
	12	0.30	11.76	45.70	42.54
	18	0.33	12.16	52.30	35.54
	24	0.36	11.22	56.82	31.96

### Causality tests

Granger causality is utilized to test causal relationships between variables in a time series [14]. The results of the test for 1982 to 2021 data are shown in Table 13. The results suggest that copper prices cause crude oil prices. The causal relationships of interest in this study are those flowing from crude oil prices to maize and copper prices. The results show that crude oil prices Granger cause neither maize prices nor copper prices.

**Table 13:** Granger causality (1982 to 2021 data)

Null hypothesis (Ho)	*χ*^2^	Prob	Result
Copper price does not cause maize price	3.972015	0.2645	Accept
Crude oil price does not cause maize price	1.213336	0.7498	Accept
Copper and crude oil prices do not cause maize price	5.102809	0.5307	Accept
Maize price does not cause copper price	1.01283	0.7981	Accept
Crude oil price does not cause copper price	4.038705	0.2573	Accept
Maize price and crude oil price do not cause copper price	5.13613	0.5265	Accept
Maize price does not cause crude oil price	1.391198	0.7076	Accept
Copper price does not cause crude oil price	17.16264	0.0007	Reject
Copper price and maize price do not cause crude oil price	18.71209	0.0047	Reject

Similarly, Granger causality test results for the 2000 to 2021 sample are shown in Table 14. Similarly, the causal relationships of interest (flowing from crude oil prices to copper prices and maize prices) showed that crude oil prices do not Granger cause maize and copper prices. Similar to the findings based on the 1982 to 2021 data, copper prices were found to Granger cause crude oil prices. They were also found to cause maize prices.

**Table 14:** Granger Causality (2000 to 2021 data)

Null Hypothesis (Ho)	χ^2^	Prob	Result
Copper price does not cause maize price	11.1761	0.0037	Reject
Crude oil price does not cause maize price	1.47234	0.4789	Accept
Copper and crude oil prices do not cause maize price	12.9388	0.0116	Reject
Maize price does not cause copper price	0.09482	0.9537	Accept
Crude oil price does not cause copper price	4.00444	0.135	Accept
Maize price and crude oil price do not cause copper price	4.72246	0.317	Accept
Maize price does not cause crude oil price	3.35078	0.1872	Accept
Copper price does not cause crude oil price	15.2488	0.0005	Reject
Copper price and maize price do not cause crude oil price	24.1016	0.0001	Reject

Tests for autocorrelation, normality and stability of the models were also done to provide reasonable assurance of the results. All the tests confirmed the necessary conditions.

## Discussion

This study has explained the effects of crude oil prices on copper prices and maize prices. The findings are that in the period 1982 to 2021, a long-run relationship exists between copper and crude oil prices (on the one hand) and maize prices (on the other). This is similar to the findings of Nwoko et al. [29] who found a long-run relationship between crude oil prices and food price volatility. The long-run relationship was found to be positive, confirming the Dutch disease theory and economic growth theory. The higher the prices of minerals (copper and crude oil), the higher the price of agriculture produce (maize in this case). A slow speed of adjustment (5.72%) to long-run equilibrium was found. This suggests that copper and crude oil prices are relatively weak policy instruments for influencing maize prices. Granger causality results also backed this up. No causal relationship was found between crude oil prices and maize prices. This is in line with Nazlioglu and Soytas [27] who also found no evidence of causality. The lack of Granger causality could be because of high labor intensity in maize production. The finding is however in contrast with Jiang et al. [21] who found that crude oil prices transmit volatility to maize prices. Variance decomposition showed that over a 24-month horizon, changes in copper prices and crude oil prices accounted for a total of about 30% of changes in maize prices. This confirms the asymmetric effect theory of mineral prices, that is, mineral prices have varying effects on food price volatility in varying contexts. For robustness, the tests were carried out on a sample of data from January 2000 to June 2021. The outcomes were more or less the same except that no cointegration relationship was found at the 5% significance level. Slight variations were noted, most likely due to the intense instability from the global financial crisis and COVID-19 pandemic in early and later years of the period 2000 to 2021.

## Conclusions

The study has explained the effects of crude oil prices on copper prices and maize price. It therefore makes a contribution to food security literature in the context of both the bottom of the pyramid (BoP) nations as well as the top ones. It also informs energy, mining, agriculture and general development policy and practice as per recommendations section. Understanding the short- and long-run relationships is essential for mining, agriculture, energy and general development policy makers and practitioners in these countries. It is also useful for bilateral aid agencies and multi-lateral aid agencies. The time series dataset used in the study consists of monthly data collected from the World Bank (WB) for the period from January 1982 to June 2021. The results indicate the existence of a long-run relationship between the two mineral prices (crude oil and copper) on the one hand and maize prices on the other. Varying short-term relationships were also found and explained using IRFs and VDFs. Granger causality flowing from crude oil prices to copper and maize prices for the period 1982 to 2021 was however not found. Recommendations are made and limitations in the study are highlighted.

## Recommendations

This study found a long-run relationship among copper prices, crude oil prices and maize prices. Mineral, energy and agriculture sector policy makers and practitioners can therefore work together to achieve long-run stability in maize prices and by extension, stability in food prices. Strategic maize reserves that create buffer inventory and smoothen supply should be encouraged. This could be coupled with increased diversification from mining to farming in order to incentivize farmers for greater food security. Strategic oil reserves should also be enhanced in order to maintain stability in the long run.

The confirmation of the Dutch disease in this study suggests that national trade policy as well as bilateral and multi-lateral trade agreements should factor in the natural resources that each party has. Countries with more mineral reserves must ensure that they get a good enough price to compensate for effects that increased mining is likely to have on other industries. Those without natural resources can intensify on provision of other goods (for instance, agriculture output such as maize).

The first limitation of this study is that the maize prices used are more representative of US prices than the rest of the world. For better generalizability, future studies could use an average of maize prices from various geographical areas of the world. In the short run, little to no association between mineral prices and maize prices is found. This suggests that other factors (say climate change) have more influence on maize prices. These could be the focus of future studies and policy decisions. The time series suggests that commodity prices experienced relatively large fluctuations in the mid-1980s, mid-2000s and early 2020. Future studies must consider focusing on expounding the effects such fluctuations had generally.

## Data Availability

The datasets generated and/or analyzed during the current study are available in the World Bank Open Data repository, https://data.worldbank.org/.

## Abbreviations

ADF: Augmented Dickey Fuller
AIC: Akaike's information criterion
BoP: Bottom of the pyramid
G20: Group twenty
FPE: Final prediction error
FOB: Free on board
HQIC: Hannan–Quinn information criterion
IRF: Impulse response function
OPEC: Organization of the Petroleum Exporting Countries
SBIC: Schwarz's Bayesian information criterion
SSA: Sub-Sahara Africa
VAR: Vector autoregression
VDF: Variance decomposition functions
VECM: Vector error correction model
WB: World Bank
WTI: West Texas Intermediate
